# Recent Emergence of *Anaplasma phagocytophilum* in Ontario, Canada: Early Serological and Entomological Indicators

**DOI:** 10.4269/ajtmh.19-0166

**Published:** 2019-10-14

**Authors:** Mark P. Nelder, Curtis B. Russell, L. Robbin Lindsay, Antonia Dibernardo, Nicholas C. Brandon, Jennifer Pritchard, Steven Johnson, Kirby Cronin, Samir N. Patel

**Affiliations:** 1 Enteric, Zoonotic and Vector-Borne Diseases, Communicable Diseases, Emergency Preparedness and Response; Public Health Ontario, Toronto, Canada;; 2 Field Studies, Zoonotic Diseases and Special Pathogens, National Microbiology Laboratory, Public Health Agency of Canada, Winnipeg, Canada;; 3 Analytic Services, Informatics, Knowledge Services, Public Health Ontario, Toronto, Canada;; 4 Public Health Ontario Laboratory, Public Health Ontario, Toronto, Canada;; 5 National Microbiology Laboratory, Public Health Agency of Canada, Winnipeg, Canada;; 6 Department of Laboratory Medicine and Pathobiology, University of Toronto, Toronto, Canada

## Abstract

Human granulocytic anaplasmosis (HGA), caused by the bacteria *Anaplasma phagocytophilum*, is transmitted to humans by blacklegged ticks (*Ixodes scapularis*) in eastern North America. To assess the emergence of *A*. *phagocytophilum* in Ontario, we analyzed patient serological and clinical data in combination with pathogen detection in blacklegged ticks from 2011 to 2017. Our sample population included all patients who had *Anaplasma* serological testing ordered by their physicians (*n* = 851). Eighty-three patients (10.8%) were *A*. *phagocytophilum* seropositive (IgG titers ≥ 1:64) and 686 (89.2%) were seronegative (IgG titers < 1:64). Applying published surveillance case definitions, we classified zero as confirmed, five as probable, and 78 as suspected cases. The percentage of seropositive patients remained generally stable at 13.6%. Seropositive patients were most often adult females, 40–59 years of age, and reported nonspecific signs and symptoms, such as fatigue, headache, and fever. Higher seropositivity rates (≥ 1.5 patients per 100,000 population) occurred in eastern and northwestern Ontario. The percentage of *A*. *phagocytophilum*-positive blacklegged ticks, through passive and active surveillance, was 0.4 and 1.1%, respectively, and increased over time. Serological and entomological indicators of *A*. *phagocytophilum* activity increased in areas of the province with established blacklegged tick populations. The risk of HGA is presently low in Ontario; however, further research is required to document the epidemiology of HGA in the province. To minimize the impact of HGA emergence in Ontario, increased awareness and education of the public and health-care providers is recommended, with consideration to making HGA a reportable infection in Ontario.

## INTRODUCTION

Human granulocytic anaplasmosis (HGA) is a tick-borne disease caused by the obligate, intracellular bacteria *Anaplasma phagocytophilum*. *Anaplasma phagocytophilum* infects granulocytes (i.e., neutrophils) and early infection, while often asymptomatic, can present as a febrile illness with nonspecific symptoms, such as arthralgia, headache, malaise, and myalgia; less common symptoms include a stiff neck, gastrointestinal complaints, and cough.^1,2^ Laboratory abnormalities in HGA patients include thrombocytopenia, leukopenia, elevated creatinine levels, anemia, and elevated hepatic transaminase levels.^2,3^ Most patients recover fully after appropriate antibiotic treatment; however, if untreated, the infection can lead to serious outcomes, such as neurological complications, opportunistic secondary infections, disseminated intravascular coagulation, organ failure, and acute respiratory distress.^3,4^ Severe illness is more common in patients older than 50 years and those with immunocompromising conditions (e.g., undergoing chemotherapy or organ transplant).^5,6^ Deaths from *A*. *phagocytophilum* infections are rare and, in the United States, case fatality rates are ≤ 1%.^4,5^ Human granulocytic anaplasmosis occurs worldwide, with the highest incidence in North America.

In eastern and central North America, including Mexico, blacklegged ticks (*Ixodes scapularis*) transmit *A*. *phagocytophilum* to humans, with symptoms appearing 5–21 days (average 7–14 days) after a tick bite.^2,7^ Although primarily a tick-borne infection, rare reports exist of blood transfusion, perinatal, and percutaneous or inhalation transmission while butchering a deer carcass (alternate modes of transmission were not ruled out in the latter two examples).^8–10^ The primary reservoirs for *A*. *phagocytophilum* likely vary locally and include rodents, such as white-footed mice (*Peromyscus leucopus*), eastern chipmunks (*Tamias striatus*), northern short-tailed shrews (*Blarina brevicauda*), and eastern gray squirrels (*Sciurus carolinensis*).^11,12^ When blacklegged ticks acquire *A*. *phagocytophilum* as larvae or nymphs, the bacteria is passed transstadially; transovarial transmission (female to egg) does not occur and larvae do not transmit the pathogen.^13^ In eastern and central North America, HGA risk is greatest wherever *A*. *phagocytophilum* is cycling in resident blacklegged tick and rodent populations and, based on the seasonality of human cases, nymphal and adult female blacklegged ticks are the life stages involved in transmission. Finally, based on the sequences of the *16S rRNA* gene, there are at least two strains of *A*. *phagocytophilum* (Ap-ha and Ap-variant 1) that circulate in North America, and these strains are detected in blacklegged ticks in varying proportions across Canada.^14,15^ The principal reservoir host of Ap-ha is the white-footed mouse, whereas the primary reservoir host of Ap-variant 1 is the white-tailed deer.^16,17^ Only Ap-ha has been implicated in causing HGA, whereas Ap-variant 1 appears not to be associated with human infection or disease.^18,19^

Human granulocytic anaplasmosis occurs in the same regions as Lyme disease (caused by *Borrelia burgdorferi*), and blacklegged ticks transmit both pathogens. In the United States, HGA incidence rates have been increasing since 2000, with higher rates in Lyme disease-endemic states of the Upper Midwest (Minnesota and Wisconsin) and Northeast (Connecticut, New York, Rhode Island, and Vermont).^6^ Asymptomatic infection with *A*. *phagocytophilum* is common. In Wisconsin, *A*. *phagocytophilum* seroprevalence in otherwise healthy adults with no history of a tick bite was 15%.^20^ In New York, *A*. *phagocytophilum* seroprevalence was 36% in asymptomatic adults with a history of a tick bite.^21^

The risk of *A*. *phagocytophilum* infection in Canada is relatively low, compared with that in the endemic regions of the United States, but the pathogen has been detected in blacklegged tick populations across Canada.^15^ Blacklegged ticks continue to increase in number and geographic distribution in Ontario, Canada, thus increasing the risk of infection from *I*. *scapularis*-associated pathogens, particularly *B*. *burgdorferi*.^22–24^ In 2009, the first locally acquired case of HGA in Canada was diagnosed in an Alberta resident.^25^ Manitoba is the only province in Canada where HGA is reportable, and approximately 12 HGA cases have been reported annually since 2015.^26^ Human granulocytic anaplasmosis is not a reportable disease in Ontario, although researchers have detected *A*. *phagocytophilum* in the province’s blacklegged ticks, dogs, white-tailed deer, and rodents.^15,23,27,28^ In 2018, the first human case of HGA acquired in Ontario was reported, emphasizing a need for enhanced *A*. *phagocytophilum* surveillance in the province.^29^ Given the presence of vector and reservoir populations, there is the need to evaluate the HGA threat in Ontario. In the absence of mandatory reporting for HGA, laboratory data are a useful tool for early detection of clinical cases and for evaluating the risk of HGA in Ontario. Here, we assess the emergence of *A*. *phagocytophilum* in Ontario by examining patient serological and clinical data in combination with pathogen testing of blacklegged ticks from 2011 to 2017.

## METHODS

### Study location.

Ontario, located in North America’s Great Lakes region (41.7°N to 56.8°N, −74.4°W to −95.2°W), is the most populous province (14 million) in Canada. Ontario’s population is concentrated in the southern portion of the province (south of 46°N), a region dominated by a moderate, humid, continental climate with a mixture of agricultural, suburban, and urban landscapes.

During the surveillance period (2011–2017), 36 public health units (PHUs) administered aspects of Ontario’s passive and active tick surveillance programs: ALG, Algoma District; BRN, Brant County; CHK, Chatham-Kent; DUR, Durham region; ELG, Elgin-St. Thomas; EOH, Eastern Ontario; GBO, Grey Bruce; HAL, Halton Regional; HAM, city of Hamilton; HDN, Haldimand-Norfolk; HKP, Haliburton-Kawartha–Pine Ridge District; HPE, Hastings and Prince Edward Counties; HUR, Huron County; KFL, Kingston-Frontenac and Lennox and Addington; LAM, Lambton; LGL, Leeds-Grenville and Lanark District; MSL, Middlesex-London; NIA, Niagara Regional; NPS, North Bay Parry Sound District; NWR, Northwestern; OTT, City of Ottawa; OXF, Oxford County; PDH, Perth District; PEL, Peel Regional; PQP, Porcupine; PTC, Peterborough County-City; REN, Renfrew County and District; SMD, Simcoe Muskoka District; SUD, Sudbury District; THB, Thunder Bay District; TOR, City of Toronto; TSK, Timiskaming; WAT, Waterloo; WDG, Wellington-Dufferin–Guelph; WEC, Windsor-Essex County; and YRK, York Regional.

### Human serological testing.

The sample population for this cross-sectional study included all patients who had *Anaplasma* serological testing ordered by their physician from January 1, 2011 through December 31, 2017. Health-care providers requesting *A*. *phagocytophilum* testing submitted whole blood or sera to Public Health Ontario (PHO), reporting the patient clinical symptoms, symptom onset date, history of a tick bite, travel history, age, gender, and residential postal code. If symptom onset date was missing, we used the date sample was taken as a proxy for estimating onset dates. Public Health Ontario sent specimens to the National Microbiology Laboratory (NML, Public Health Agency Canada, Winnipeg, Manitoba) for *A*. *phagocytophilum* IgG serology. The NML performed an indirect immunofluorescence assay (IFA) using the Focus *A. phagocytophilum* IFA IgG kit (DiaSorin Molecular, Cypress, CA), according to the manufacturer’s instructions. Briefly, we added test sera diluted 1:64 in phosphate buffer saline to wells of IFA slides precoated with HGE-1 strain-infected HL-60 cells and incubated for 30 minutes at 37°C. Following incubation, we washed the slides to remove unbound serum. We added a fluorescein-labeled antibody to human IgG to each well and incubated the slides for 30 minutes at 37°C. The slides were then washed, air-dried, mounted, and examined using fluorescence microscopy.

We obtained semiquantitative endpoint titers by testing serial dilutions of positive sera, where the reciprocal of the highest serum dilution exhibiting fluorescence of the morulae was considered the serum endpoint titer. As per manufacturer recommendations, we report IgG titers ≥ 1:64 as positive results. Single IgG serum endpoint titers ≥ 1:64 were suggestive of infection at an undetermined time and may be indicative of either past infection or early response to a recent infection. A 4-fold or greater increase in IgG titer between two serum samples drawn 2 to 4 weeks apart and tested in parallel was considered evidence of recent or current infection by *A*. *phagocytophilum*, based on existing case definitions.^30,31^

### Surveillance case definitions.

In most circumstances where a pathogen is reportable to public health, assessing disease risk is relatively straightforward. However, in an area where a pathogen is potentially emerging and the disease is not yet reportable, an interim surveillance plan is required. Our approach included examination of patient serological and clinical data in combination with pathogen testing of blacklegged ticks. For classifying seropositive patients with anaplasmosis, we used case definitions developed by the Manitoba Public Health Branch and the U.S. CDC.^30,31^ Surveillance case definitions, as opposed to clinical case definitions, were used as available clinical data were limited to information provided on laboratory requisitions.

### Passive tick surveillance.

Briefly, PHO identifies ticks submitted by the public through health-care providers (e.g., clinician offices) or through PHUs and then sends blacklegged ticks to the NML for pathogen detection (see upcoming section).^23,32^ Data captured included the submitter’s city of residence, age, gender, date of tick submission, life stage and/or sex of tick collected, and the submitter’s travel history. If the location of tick acquisition was not specified, we used the city of residence assuming that the most likely exposure location was near or in the submitter’s city of residence.^33^ In 2014, the PHUs of EOH, KFL, and LGL ceased accepting tick submissions directly from the public at their public health offices; however, health-care providers could still submit ticks from patients.

### Active tick surveillance.

The objective of active surveillance, like passive surveillance, is to identify established blacklegged tick populations and to assess Lyme disease risk.^34,35^ A risk area is defined as a location where at least one blacklegged tick is collected during spring (April and May) and fall sampling events (October and November) of the same year; a sampling event is defined as at least 3 person-hours of drag sampling at one location.^36^ Blacklegged ticks collected through the active tick surveillance program from 2015 to 2017 were sent to the NML for identification and pathogen detection (described in the next section). Data for each tick collected included stage, sex, collection location, and date of collection.

### Testing blacklegged ticks for pathogens.

Blacklegged ticks submitted to the NML through active and passive tick surveillance are routinely tested for DNA or RNA of *A*. *phagocytophilum*, *Babesia microti*, a variety of *Borrelia* species including *B*. *burgdorferi*, and Powassan encephalitis virus by real-time polymerase chain reaction (PCR) as previously described.^37,38^ Briefly, for the detection of *A*. *phagocytophilum* DNA in ticks, we used Qiagen^®^ DNeasy 96 tissue kits (Qiagen Inc., Mississauga, ON) for DNA extraction as per the manufacturer’s instructions. We eluted DNA in 200 μL of AE buffer and stored at −80°C before use. We used a duplex real-time PCR assay to screen the samples for *A*. *phagocytophilum* by targeting the *msp2* gene.^39^

We monitored each round of DNA extractions for cross-contamination by including at least two samples consisting only of nuclease-free water. Synthetic double-stranded DNA controls (Integrated DNA Technologies, Skokie, IL) for *Anaplasma* were included as positive controls in each PCR run, whereas no-template controls consisting of master mix only served as negative controls. In addition, our positive control DNA for *A*. *phagocytophilum* was an equine isolate (MN-93, courtesy of Tim Kurtti, University of Minnesota, MN) that had been propagated in HL-60 promyelocytic cell line (ATCC CCL-240).

### Genotyping of *A*. *phagocytophilum* by single-nucleotide polymorphism (SNP) real-time PCR.

Differentiation of Ap-ha and Ap-variant 1 strains of *A*. *phagocytophilum* was accomplished using a TaqMan^®^ real-time allelic discrimination assay based on a nucleotide difference at the 5′ end of the *16S rRNA* gene sequence.^15^ Briefly, real-time PCR was performed with a master mix consisting of 12.5 μL of TaqMan Universal Master Mix (Applied Biosystems, Foster City, CA), 1.25 μL of 20× custom TaqMan SNP genotyping assay, and 6.25 μL nuclease-free water, followed by 5 μL of sample DNA, for a total reaction volume of 25 μL. Amplification was performed on a 7,500 Sequence Detection System (Applied Biosystems) using universal thermocycling conditions of 2 minutes at 50°C, 10 minutes at 95°C for AmpliTaq Gold^®^ activation, 40 cycles of 95°C for 15 seconds, and 60°C for 1 minute.

We performed SNP genotyping analysis using SDS v2.0.5 (Applied Biosystems). We used a multicomponent algorithm to calculate the distinct signal contribution of each allele and generated an allelic discrimination plot for visual representation of the distribution of alleles.

### Statistical analyses and mapping.

We calculated PHU and provincial rates per 100,000 population of seropositive patients and positive blacklegged tick submissions using population data and projections from Statistics Canada via IntelliHEALTH Ontario as denominators (extracted October 19, 2017). Excel v14.0 (Microsoft, 2010, Redmond, WA) was used for obtaining descriptive statistics, tests of independence (i.e., chi-squared test), and differences in means (i.e., analysis of variance). We created maps using Esri ArcGIS v10.3 (Esri, 2014, Redlands, CA), using manual classification methods to classify PHU rates.

### Ethics statement and data availability.

This article reports on routine surveillance activities, and therefore, approval from the research ethics committee was not required. Information about PHO’s data request process is available online at https://www.publichealthontario.ca/en/About/Pages/data.aspx.

## RESULTS

### Human serology.

**Figure 1.**
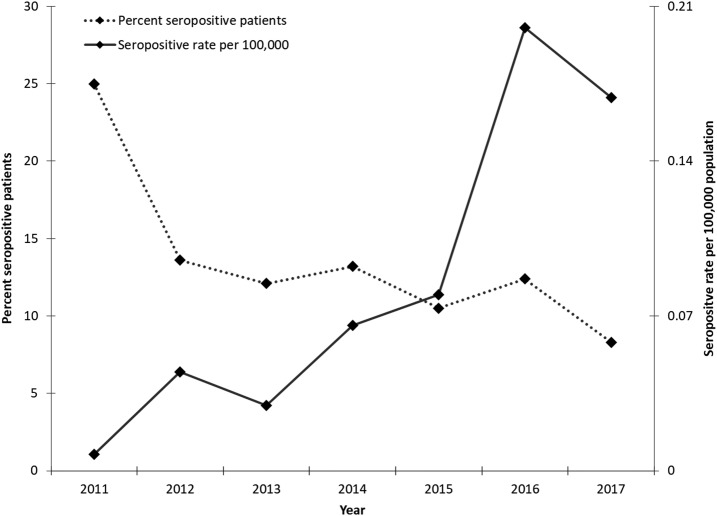
Percent *Anaplasma phagocytophilum*-seropositive patients and seropositive rates per 100,000 population in Ontario, Canada (2011–2017).

Sera from 851 patients were tested for *A*. *phagocytophilum* antibodies from 2011 through 2017 (representing 943 specimens tested); 97 patients (11.4%) were seropositive (IgG titer ≥ 1:64) and 754 (88.6%) were seronegative (IgG titer < 1:64). Fourteen seropositive patients had traveled outside of Ontario during the incubation period (United States [*n* = 5], Europe [*n* = 4], Central and South America [*n* = 4], Africa [*n* = 2], Asia [*n* = 1], and Manitoba [*n* = 1]; includes travel to multiple countries). In addition, 68 seronegative patients traveled outside of Ontario during the incubation period (United States [*n* = 23], Europe [*n* = 22], Central and South America/Caribbean [*n* = 16], Asia/Oceania [*n* = 5], Africa [*n* = 3], Nova Scotia [*n* = 3], Australia [*n* = 2], and British Columbia [*n* = 1]; includes travel to multiple countries). We excluded travel-related patients from further analyses. We performed analysis on a final dataset of 769 non-travel patients, including 83 seropositive (10.8%) and 686 seronegative (89.2%) patients (Table 1). None of Ontario’s seropositive patients met Manitoba’s confirmed or probable case definitions. After applying the U.S. CDC case definition, zero cases met the confirmed classification, five probable, and 78 suspected. Five of 83 (6.0%) seropositive patients had paired acute and convalescent sera tested (one with a 4-fold increase in IgG titers, but with no signs or symptoms reported). The average annual seropositivity (no. of seropositive patients/total patients tested) remained near 13.6%, with an annual average seropositive rate of 0.090 per 100,000 population increasing over time from 0.0074 (2011) to 0.18 per 100,000 (2017) (Figure 1).

**Table 1:** Patient-level *Anaplasma phagocytophilum* IgG serological results and human granulocytic anaplasmosis case classifications, Ontario, Canada (2011–2017)

Acute *A*. *phagocytophilum* IgG titer	No. of sera samples	Human granulocytic anaplasmosis case classification*		
		Confirmed	Probable	Suspected
< 1:64	686	NA	NA	NA
1:64	43	0	2	41
1:128	24	0	1	23
1:256	7	0	0	7
≥ 1:512	9	0	2	7
Total	769	0	5	78

NA = not applicable.

* Case classification based on U.S. CDC case definitions.^31^

Symptom onset (3.6%; *n* = 3) and sample taken (71.1%; *n* = 59) dates were available for seropositive patients. Most seropositive patients had an estimated onset in August (14.5%; *n* = 9) and September (19.4%; *n* = 12). Thirty-four (41.0%) seropositive patients reported at least one sign or symptom, including fatigue (50.0%), headache (44.0%), and fever (20.6%) (Table 2).

**Table 2:** Demographics and clinical presentation of *Anaplasma phagocytophilum*-seropositive and seronegative patients, Ontario, Canada (2011–2017)

Demographics and clinical presentation	No. of seropositive patients (%) (*n* = 83)	No. of seronegative patients (%) (*n* = 686)
Gender		
Male	33 (39.8)	240 (35.0)*
Female	50 (60.2)	430 (62.7)
Unknown	0 (0.0)	16 (2.3)
Age (years)		
Mean ± SE	42.7 ± 1.73	42.5 ± 0.65†
Age group		
0–9	2 (2.4)	22 (3.2)
10–19	4 (4.8)	52 (7.6)
20–29	13 (15.7)	88 (12.8)
30–39	14 (16.9)	126 (18.4)
40–49	18 (21.7)	132 (19.2)
50–59	18 (21.7)	152 (22.2)
60–69	10 (12.0)	74 (10.8)
70–79	3 (3.6)	34 (5.0)
80–89	0 (0.0)	3 (0.4)
Unknown	1 (1.2)	3 (0.4)
Signs and symptoms‡		
Fatigue	17 (50.0)	79 (22.9)
Headache	15 (44.0)	130 (37.7)
Fever	7 (20.6)	103 (29.9)
Gastrointestinal complaints	4 (11.8)	29 (8.4)
Arthralgia	3 (8.8)	27 (7.8)
Rash (non-erythema migrans)	3 (8.8)	26 (7.5)
Elevated liver enzymes	1 (2.9)	4 (1.2)
Weight loss	1 (2.9)	3 (0.9)
Dizziness	1 (2.9)	1 (0.3)
Malaise	1 (2.9)	4 (1.2)
Respiratory complaints	0 (0.0)	19 (5.5)
Encephalitis/meningitis	0 (0.0)	16 (4.6)
Myalgia	0 (0.0)	5 (1.4)
Confusion	0 (0.0)	3 (0.9)
Chills	0 (0.0)	2 (0.6)
Anemia	0 (0.0)	1 (0.3)
Acute hepatitis	0 (0.0)	1 (0.3)
Jaundice	0 (0.0)	1 (0.3)
No. of patients reporting ≥ one sign or symptom	34	345

* Ratio of female to male seropositive and seronegative patients (χ^2^ = 0.50, *P* = 0.48).

† Mean of age for seropositive and seronegative patients (*F*_1, 763_ = 0.0066, *P* = 0.94).

‡ Percentages do not total 100%, as multiple symptoms can be reported for each patient.

There was no difference in the percentage of female seropositive (60.2%; *n* = 50) and seronegative patients (62.7%; *n* = 430) (χ^2^ = 0.50; *P* = 0.48), or in the mean age of seropositive (42.7 ± 1.73 years) and seronegative patients (42.5 ± 0.65 years) (*F*_1, 763_ = 0.0066; *P* = 0.94) (Table 2). The number of seropositive patients peaked in 40- to 59-year-olds.

**Figure 2.**
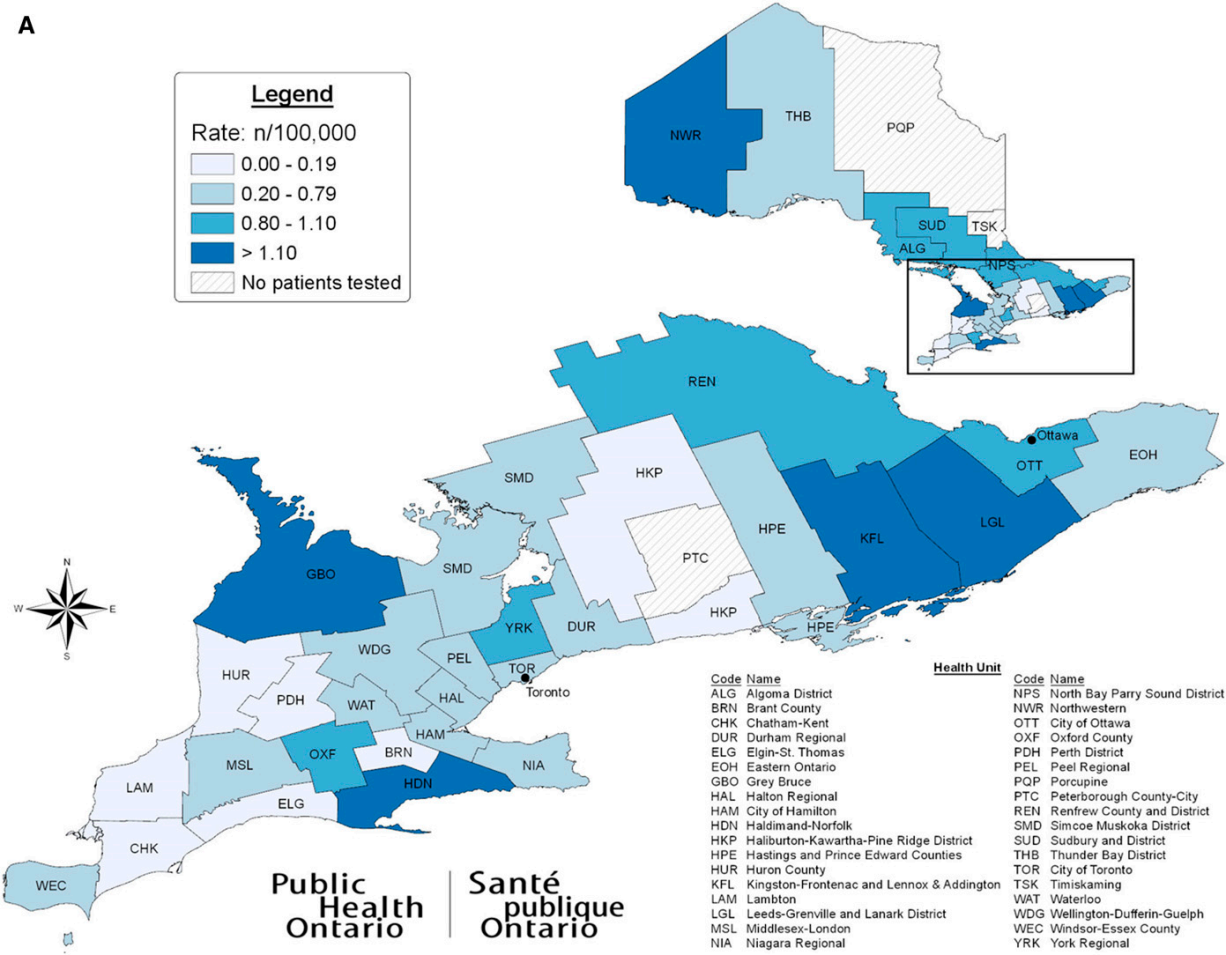

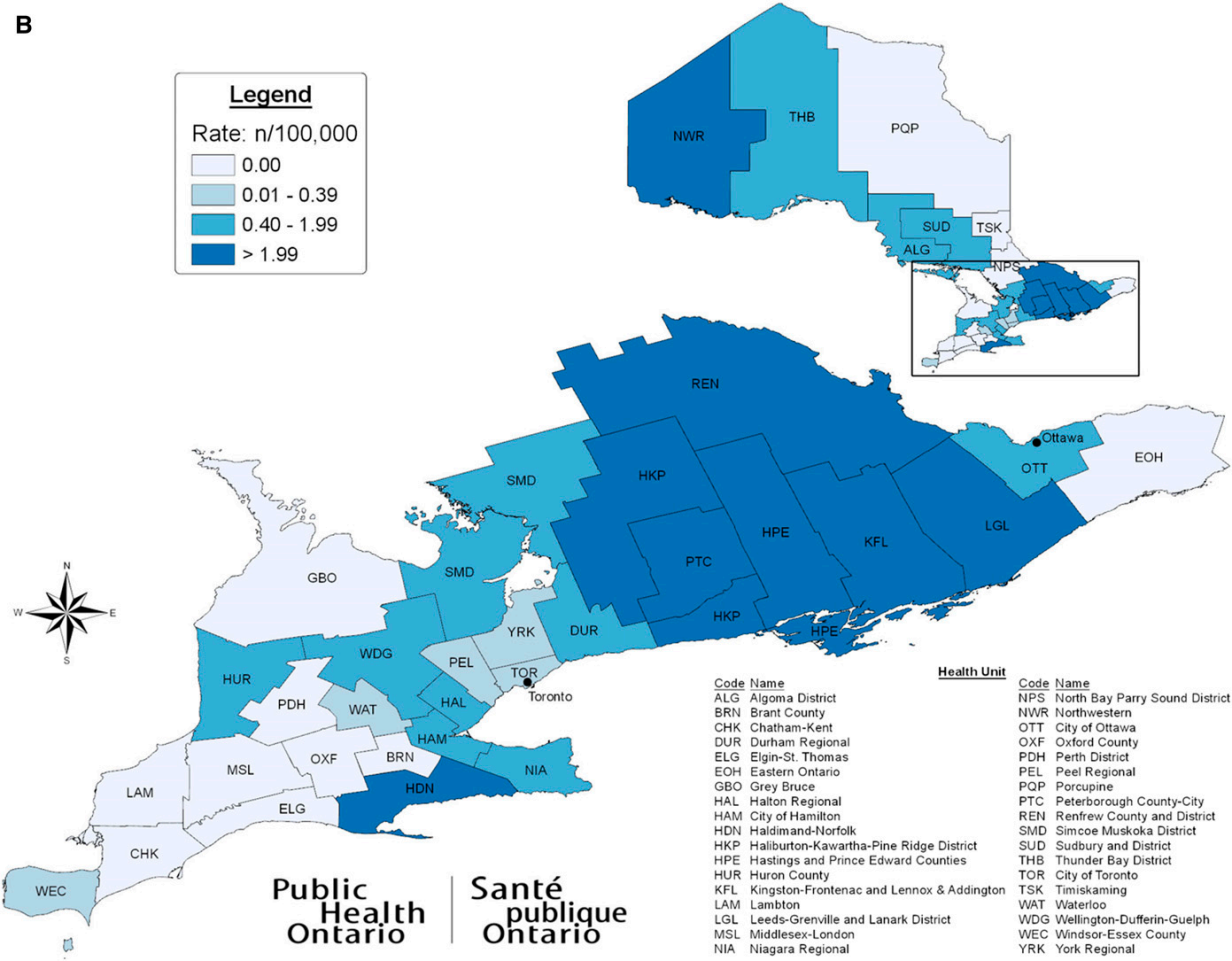
*Anaplasma phagocytophilum* activity by public health unit, Ontario, Canada (2011–2017). (**A**) *Anaplasma phagocytophilum*-seropositive patients per 100,000 population. (**B**) *Anaplasma phagocytophilum*-positive blacklegged tick submissions per 100,000 population (passive surveillance).

We detected seropositive patients in 26 of 33 PHUs where at least one patient was tested. Higher seropositive rates were from NWR (3.7 patients per 100,000), HDN (1.8 per 100,000), and KFL (1.5 per 100,000) (Supplemental Table 1, Figure 2).

### Passive blacklegged tick surveillance.

**Figure 3.**
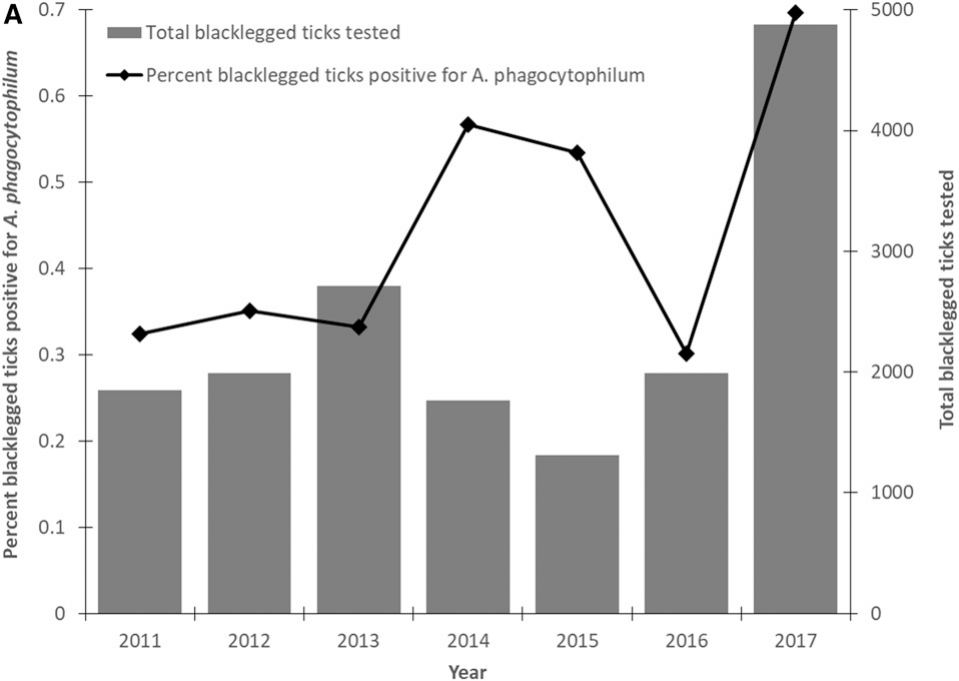

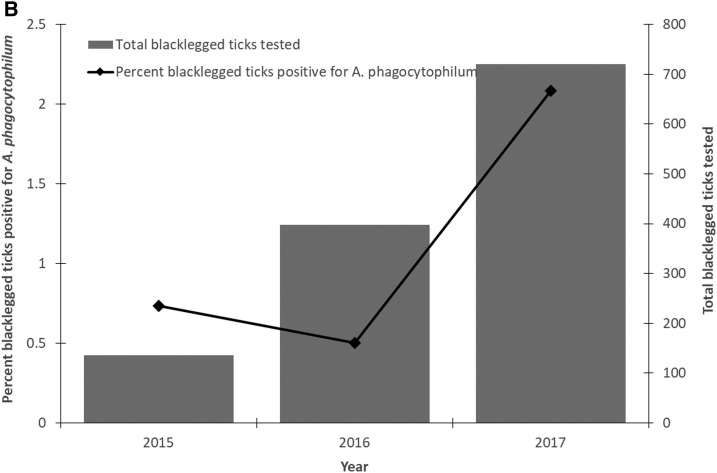
Percent blacklegged ticks positive for *Anaplasma phagocytophilum* using (**A**) passive (2011–2017) and (**B**) active (2015–2017) surveillance in Ontario, Canada.

From 2011 to 2017, 79 of 16,494 blacklegged ticks collected from passive surveillance were positive for *A*. *phagocytophilum* (Supplemental Table 1). The average annual percent positivity in ticks was 0.4% and increased over time (Figure 3). Blacklegged ticks tested included 15,363 adults (13,940 females, 273 males, and 58 mixed-sex pools), 1,084 nymphs, 37 larvae, and 10 mixed-stage pools. Seventy-four of 79 positive ticks were adults (69 females, four males, and one mixed-sex pool) and five were nymphs. We tested 53 of the positive ticks using the SNP assay and 26.4% (*n* = 14) were infected with the Ap-ha strain and 73.6% (*n* = 39) with Ap-variant 1.

We detected at least one positive tick in 24 of 36 PHUs (Supplemental Table 1). Higher positive tick submissions occurred in HKP (4.4 per 100,000), PTC (3.6 per 100,000), and HPE (3.1 per 100,000) (Figure 2). We detected the Ap-ha strain in three ticks from THB; two ticks from WDG; and one tick each in DUR, HAL, LGL, NIA, NWR, REN, TOR, WAT, and YRK.

### Active blacklegged tick surveillance.

From 2015 to 2017, 18 of 1,252 blacklegged ticks collected during active surveillance were positive for *A*. *phagocytophilum* (Supplemental Table 1). The average annual percent positivity was 1.1% and increased over time (Figure 3). Ticks tested included 1,147 adults (525 females and 622 males) and 105 nymphs. All positive ticks were adults (eight females and 10 males). We tested 16 of the positive ticks using the SNP assay and 18.7% (*n* = 3) were infected with the Ap-ha strain and 81.3% (*n* = 13) with Ap-variant 1.

We detected at least one *A*. *phagocytophilum*-positive blacklegged tick in six of 16 (37.5%) PHUs (which conducted active surveillance), with highest percent positivity in NWR (7.6%; 8/106). We detected the Ap-ha strain in two ticks from NWR and one tick from LGL.

## DISCUSSION

*Anaplasma phagocytophilum* percent seropositivity was stable from 2011 through 2017; however, while low, rates of seropositive patients in the population rose. The stable seropositivity with increased seropositive rate in the human population was likely the result of increased numbers of tests being performed in the province. Applying the U.S. CDC surveillance case definitions to 83 seropositive patients in Ontario, we classified zero as confirmed (one had a 4-fold increase in IgG titers, but with no clinical signs or symptoms reported), five as probable, and 78 as suspected cases. We expected seropositive rates for HGA to be low in Ontario because it is not a reportable disease and clinical suspicion for cases presenting with compatible symptoms is likely to be low. In addition, because we performed serology without PCR testing, we could have missed cases. In addition, we would miss clinical cases that did not meet the surveillance case definition. In the United States (2008–2012), public health officials classified 99% of confirmed HGA cases based on PCR-positive specimens, compared with 0.5% of confirmed cases based on seroconversion evidence.^6^

In 2018, researchers reported the first case of a locally acquired *A*. *phagocytophilum* infection (2017 infection) in Ontario. The patient presented with fever, headache, nausea, vomiting, thrombocytopenia, and leukopenia, with evidence of a 4-fold increase in IgG titers.^29^ The clinical signs and symptoms of Ontario’s seropositive patients are similar to those for HGA cases reported elsewhere; however, the clinical spectrum of Ontario’s patients is difficult to characterize, given the lack of available clinical information, which is based solely on information provided on laboratory requisitions.

We expect a continued rise in patients exposed to *A*. *phagocytophilum* in Ontario, similar to increasing HGA incidence rates in the United States, from 2.0 (2000–2007) to 6.3 per million person-years (2008–2012).^5,6^ In Minnesota, HGA incidence rates have increased from 1.3 (2003) to 11.6 per 100,000 (2017).^40^ In Manitoba, confirmed and probable cases have increased from four in 2015 to 21 in 2018.^26^ In Québec, where HGA is not reportable, annual seropositivity has remained stable at approximately 17% (2012–2016).^41,42^ The identification of potential HGA cases in PHUs where the testing was performed, coupled with activity in neighboring jurisdictions in Canada and the United States, highlights the risk of HGA in Ontarians exposed to infectious blacklegged ticks.

Seropositive rates were highest in PHUs with established blacklegged tick populations, such as NWR (3.7 seropositive patients per 100,000), HDN (1.8 per 100,000), and KFL (1.5 per 100,000).^34,38^ *Anaplasma phagocytophilum* activity in northwestern Ontario and neighboring Manitoba (including pathogen positivity in ticks) is potentially higher because blacklegged tick phenology (larvae and nymph synchrony) is unique in the Upper Midwest.^43^ We cannot rule out that higher seropositivity rates in certain PHUs are, at least in part, caused by higher physician HGA awareness in areas with established vector populations. For seropositive patients, we often do not know the exposure location; therefore, some caution must be used when interpreting the distribution of seropositive patients.

The number of *A*. *phagocytophilum*-infected blacklegged ticks is gradually increasing in Ontario, likely because of a combination of increased pathogen prevalence in reservoirs and increased testing of ticks. We detected *A*. *phagocytophilum*-infected ticks in areas where we would expect emergence, specifically PHUs with established blacklegged tick populations and relatively higher Lyme disease incidence.^24,44^ We must note that the opportunity to detect *A*. *phagocytophilum* in blacklegged ticks is lower in PHUs that have stopped passive tick submissions (i.e., EOH, KFL, and LGL); however, this is overcome by active surveillance in these PHUs. A study of blacklegged ticks from passive surveillance in Ontario (2007–2010) noted that 0.3% were positive for *A*. *phagocytophilum*, similar to the 0.4% reported here.^15^ In Québec’s passive blacklegged tick surveillance, *A*. *phagocytophilum* percent positivity was higher than that in Ontario and ranged from 1.1% (2014) to 1.9% (2016); for active surveillance, the percent positivity was lower than that in Ontario at 0.6% (2016).^41,42^

In addition to human and entomological indicators of HGA emergence in Ontario, veterinary indicators exist as well. Twenty-four blacklegged ticks collected from dogs in Ontario were positive for *A*. *phagocytophilum* from 2011 through 2017 (L. R. Lindsay, unpublished data); *A*. *phagocytophilum* seroprevalence in Ontario dogs is low (< 2%; 2008–2010, 2012).^45^ In 2015, an equine granulocytic anaplasmosis case was reported from eastern Ontario.^46^

The increasing activity of *A*. *phagocytophilum* in Ontario is linked to the increasing numbers and geographic distribution of blacklegged ticks, placing more of the population at risk of HGA. Climate change is contributing to the expanding range of blacklegged ticks into southern Canada, and increases in the mean annual degree days above 0°C is a crucial factor responsible for this northward advance.^47–49^ In addition, *A*. *phagocytophilum* can increase blacklegged tick survival under cold conditions by upregulating the *I*. *scapularis* antifreeze glycoprotein.^50^ In addition, migratory birds can spread *A*. *phagocytophilum*-infected blacklegged ticks from the United States to Canada.^51^ Expanding blacklegged tick populations will continue to put Ontarians at risk of *A*. *phagocytophilum* infection.

Blacklegged ticks infected with *A*. *phagocytophilum* in our study appear to be more frequently infected with the nonpathogenic strain (Ap-variant 1) rather than the human pathogenic strain (Ap-ha), consistent with earlier studies in Ontario; e.g., a study of Ontario (2007–2010) *A*. *phagocytophilum*-positive blacklegged ticks showed 8.3% were positive for Ap-ha.^15^ A detailed study in the Thousand Islands region of Ontario showed all *A*. *phagocytophilum*-positive blacklegged ticks (*n* = 34) contained Ap-variant 1.^27^ In the current study, 19–26% of Ontario’s *A*. *phagocytophilum*-infected blacklegged ticks collected by passive or active surveillance were infected with Ap-ha, and it appears this strain is becoming more prevalent.

In addition to caveats already discussed, we note additional limitations to our study. The prevalence of *A*. *phagocytophilum* in blacklegged ticks is likely underestimated, as passive and active tick surveillance targets adult tick specimens, underrepresenting a major stage responsible for transmission (i.e., nymph). The absence of symptom onset dates (> 96% missing) in the submitted data made it difficult to estimate time of blacklegged tick exposure. Estimating onset dates is further complicated because, at least in some cases, HGA patients may have elevated IgG titers for months to years. Low awareness by health-care providers likely contributed to lower testing volumes and missed laboratory diagnoses. In addition, we likely missed seropositive patients if sera testing occurred in the first week of illness, as IFA IgG is not sensitive during this period.^52^ In HGA-endemic regions, acute IgG titers ≥ 1:512 are indicative of active infection, meaning that at least nine of the Ontario seropositive patients likely had an active infection.^2^ Recognizing limitations in IgG serology, possible lack of clinical awareness, and lack of reportability, our surveillance can be improved by encouraging submission of convalescent sera, offering PCR testing for acute HGA cases, and ensuring better completion of data elements on test requisitions (e.g., symptom onset dates and clinical signs and symptoms).

The risk of *A*. *phagocytophilum* infection in Ontario is currently low; however, we expect this risk to increase. Enhancing HGA awareness among the public and health-care providers is warranted in Ontario, which, in part, can be improved by making HGA a provincially reportable disease.

## Supplemental table

Supplemental materials
