# Supplementary material for: Recent Emergence of Anaplasma phagocytophilum in Ontario, Canada: Early Serological and Entomological Indicators
Source: Am J Trop Med Hyg. 2019 Oct 14;101(6):1249–58. doi: 10.4269/ajtmh.19-0166 (PMC6896876; doi:10.4269/ajtmh.19-0166)
Supplement: Supplementary file 1 [file tpmd190166.SD1.pdf]

SUPPLEMENTAL TABLE 1. Summary *A. phagocytophilum* activity in humans and ticks by public health unit: Ontario, Canada (2011–2017)

Public health unit	**Percent positive blacklegged ticks (no. seropositive/*n*)**		Positive tick submission rate per 100,000 population	**Percent seropositive patients (no. seropositive/*n*)**	Seropositive patient rate per 100,000 population
	Passive surveillance	Active surveillance*			
ALG	3.4 (1/29)	NAS	0.9	14.3 (1/7)	0.9
BRN	0.0 (0/130)	NAS	0.0	0.0 (0/2)	0.0
CHK	0.0 (0/95)	NAS	0.0	0.0 (0/2)	0.0
DUR	0.6 (4/656)	0.0 (0/40)	0.7	2.6 (1/39)	0.1
ELG	0.0 (0/134)	NAS	0.0	0.0 (0/3)	0.0
EOH	0.0 (0/738)	1.3 (1/80)	0.0	11.1 (1/9)	0.5
GBO	0.0 (0/151)	NAS	0.0	8.3 (2/5)	1.2
HAL	0.4 (1/266)	NAS	0.4	11.4 (4/35)	0.7
HAM	1.1 (3/268)	0.0 (0/13)	0.5	8.3 (4/48)	0.7
HDN	0.5 (6/1,289)	NAS	5.4	40.0 (2/5)	1.8
HKP	0.6 (6/1015)	0.0 (0/105)	4.4	0.0 (0/11)	0.0
HPE	0.4 (5/1,425)	NAS	3.0	9.1 (1/11)	0.6
HUR	2.8 (1/36)	NAS	1.7	0.0 (0/1)	0.0
KFL	0.4 (8/2,044)	0.6 (1/164)	2.5	13.6 (3/22)	1.5
LAM	0.0 (0/126)	0.0 (0/35)	0.0	0.0 (0/8)	0.0
LGL	0.3 (8/3,014)	1.3 (4/310)	2.9	7.7 (2/26)	1.2
MSL	0.0 (0/145)	NAS	0.0	10.0 (1/10)	0.2
NIA	0.5 (2/436)	1.3 (1/78)	0.4	8.1 (3/37)	0.7
NPS	0.0 (0/45)	NAS	0.0	25.0 (1/4)	0.8
NWR	1.2 (2/162)	7.6 (8/106)	2.5	60.0 (3/5)	3.7
OTT	0.3 (4/1,238)	0.0 (0/26)	0.5	11.6 (8/69)	0.8
OXF	0.0 (0/121)	NAS	0.0	25.0 (1/4)	0.9
PDH	0.0 (0/22)	NAS	0.0	0.0 (0/3)	0.0
PEE	1.1 (2/175)	0.0 (0/4)	0.1	9.4 (3/32)	0.2
PQP	0.0 (0/13)	NAS	0.0	NPT	NPT
PTC	1.3 (5/400)	NAS	3.5	NPT	NPT
REN	0.9 (3/341)	0.0 (0/9)	2.8	14.3 (1/7)	0.9
SMD	0.5 (2/392)	0.0 (0/8)	0.5	9.7 (3/31)	0.5
SUD	1.6 (1/61)	NAS	0.5	25.0 (2/8)	1.0
THB	1.7 (3/178)	0.0 (0/1)	1.9	10.0 (1/10)	0.6
TOR	0.5 (3/670)	1.2 (3/255)	0.1	9.4 (18/192)	0.6
TSK	0.0 (0/16)	NAS	0.0	0.0 (0/1)	NPT
WAT	0.5 (1/190)	NAS	0.2	13.0 (3/23)	0.5
WDG	3.4 (3/88)	NAS	1.0	5.9 (1/17)	0.3
WEC	1.0 (1/102)	NAS	0.2	16.7 (2/12)	0.5
YRK	1.4 (4/279)	0.0 (0/14)	0.3	15.3 (11/72)	0.9
**Provincial average (*n*/*N*) or rate**	0.5 (79/16,494)	1.4 (18/1,252)	0.6	10.8 (83/769)	0.6

ALG, Algoma District; BRN, Brant County; CHK, Chatham-Kent; DUR, Durham Regional; ELG, Elgin-St. Thomas; EOH, Eastern Ontario; GBO, Grey Bruce; HAL, Halton Regional; HAM, City of Hamilton; HDN, Haldimand-Norfolk; HKP, Haliburton-Kawartha-Pine Ridge District; HPE, Hastings and Prince Edward Counties; HUR, Huron County; KFL, Kingston-Frontenac and Lennox & Addington; LAM, Lambton; LGL, Leeds-Grenville and Lanark District; MSL, Middlesex-London; NAS = no active surveillance performed; NIA, Niagara Regional; NPS, North Bay Parry Sound District; NPT = no patient sera tested; NWR, Northwestern; OTT, City of Ottawa; OXF, Oxford County; PDH, Perth District; PHU, public health unit; PEL, Peel Regional; PHO, Public Health Ontario; PQP, Porcupine; PTC, Peterborough County-City; REN, Renfrew County and District; SMD, Simcoe Muskoka District; SE, standard error; SUD, Sudbury and District; THB, Thunder Bay District; TOR, City of Toronto; TSK, Timiskaming; WAT, Waterloo; WDG, Wellington-Dufferin-Guelph; WEC, Windsor-Essex County; YRK, York Regional

*Active tick surveillance was conducted only from 2015 to 2017
